# MIND-BASED AND MOVEMENT-BASED MIND-BODY INTERVENTION FOR CHINESE OLDER PEOPLE WITH DEPRESSION

**DOI:** 10.1093/geroni/igz038.1002

**Published:** 2019-11-08

**Authors:** Sunny H W Chan, Herman H M Lo, Chong Ho Yu

**Affiliations:** 1 The Hong Kong Polytechnic University, Hong Kong, Hong Kong; 2 Azusa Pacific University, Azusa, California, United States

## Abstract

Mind-body intervention has been well established as an alternative psychosocial intervention for managing depression. Mindfulness-based intervention (MBI) and health qigong (HQ) are two common forms of mind-body intervention which share the common focus on breathing. However, they may represent two distinct approaches with different mechanisms. MBI focuses more on mind-based practices whereas HQ may focus predominantly on body-based movement practices. Thus, a large research gap in comparing the unique therapeutic effects of mind-based and movement-based health practices on alleviating depression among older people is worthy of further investigation. A total of 45 community-dwelling Chinese older adults aged 60 or above with symptoms of clinical depression were recruited. They were randomly assigned to three different groups, including an MBI group, a HQ group, and a waitlist control (WLC) group. Comparisons were made before and after 8-week interventions. Regarding the primary outcome, the effect sizes between the MBI and WLC groups, as well as between the HQ and WLC groups, were reasonably large (Hedges’ g = 1.338 and 0.725, respectively), yet the effect size between the MBI and HQ groups was moderate (Hedges’ g = 0.325). Specifically, participants in the MBI group showed more improvements on perceived stress, self-efficacy, and mental health, whereas participants in the HQ group showed relatively better performance regarding interoception and physical mobility. Findings from this research demonstrate the unique therapeutic effects of mind-based and movement-based interventions on alleviating depression among older people. The application of two distinct forms of mind-body intervention in a Chinese context is discussed.

